# Assessment of the Impact of Comorbidities on Outcomes in Non-ST Elevation Myocardial Infarction (NSTEMI) Patients: A Narrative Review

**DOI:** 10.7759/cureus.65568

**Published:** 2024-07-28

**Authors:** Bryan Felix, Fawaz Aldoohan, Hansanee U Kadirage, Sethulakshmi Keelathara Sajeev, Maryam Kayani, Mohamed Abdelsalam Ibrahim Hag Saeed, Sruthi Vempatapu, Khadija Nasim, Harini Pendem, Annia P Armenta, Zahra Nazir

**Affiliations:** 1 Medical Student, Avalon University School of Medicine, Los Angeles, USA; 2 Internal Medicine, American Academy of Research and Academics, Delaware, USA; 3 Medicine, Kathmandu University, Kathmandu, NPL; 4 Cardiology, Tbilisi State Medical University, Tbilisi, GEO; 5 Cardiology, Shifa College of Medicine, Shifa Tameer-e-Millat University, Islamabad, PAK; 6 Emergency Medicine, Dr. Sulaiman Al-Habib Medical Group, Riyadh, SAU; 7 Internal Medicine, Nandamuri Taraka Rama Rao (NTR) University of Health Sciences, Hyderabad, IND; 8 Medicine, Jinnah Sindh Medical University, Karachi, PAK; 9 Internal Medicine, Chalmeda Anand Rao Institute of Medical Sciences, Karimnagar, IND; 10 Internal Medicine, Universidad Autónoma de Guadalajara, Guadalajara, MEX; 11 Internal Medicine, Combined Military Hospital (CMH), Quetta, PAK

**Keywords:** copd (chronic obstructive pulmonary disease), ckd(chronic kidney disease), diabete mellitus, obesity, non-st segment elevation myocardial infarction (nstemi)

## Abstract

Non-ST-segment elevation myocardial infarction (NSTEMI) is associated with significant morbidity and mortality, occurring when the heart’s need for oxygen cannot be met. It is defined by elevated cardiac biomarkers without ST-segment elevation and often carries a poorer prognosis than most ST-segment elevation events. NSTEMI usually results from severe coronary artery narrowing, transient occlusion, or microembolization of thrombus/atheromatous material. Patients with NSTEMI often have multiple comorbidities, which can worsen their prognosis and complicate treatment. This study aims to investigate the impact of comorbidities such as hypertension (HTN), diabetes mellitus (DM), chronic obstructive pulmonary disease (COPD), obesity, dyslipidemia, and smoking on patients with NSTEMI. The prevalence of each comorbidity is examined individually within the NSTEMI population to provide a clearer picture of how frequently these conditions co-occur with NSTEMI and how they affect the established NSTEMI treatment protocols.

This paper sheds light on the interaction between NSTEMI and commonly associated comorbidities through a comprehensive literature review and data analysis. This is critical for optimizing clinical decision-making and enhancing patient care, ultimately improving outcomes in this high-risk patient population.

## Introduction and background

Cardiovascular disease (CVD) is a leading cause of mortality worldwide [1]. Non-ST-Elevation myocardial infarction (NSTEMI) accounts for about 70% of all acute coronary syndrome (ACS) cases. Symptoms typically include substernal chest pain radiating to the jaw or left arm, caused by inadequate blood flow [2]. Common causes include atherosclerosis, vasospasm, or coronary embolism. The development of atherosclerosis is influenced by individual risk factors such as hypertension (HTN), hyperlipidemia, diabetes, and smoking. Atherosclerosis progresses over many decades until clinically detectable [3].

In the United States, comorbidities such as diabetes and HTN are exceptionally prevalent, affecting a significant portion of the population. According to the American Diabetes Association (2021), 38.4 million Americans, or 11.6% of the population, have diabetes. The American Heart Association (AHA) reports that approximately 47% of adults in the US have HTN.

Numerous conditions interfere with oxygen delivery to cells, including HTN and diabetes, causing vascular endothelial dysfunction [4]. This increases the workload on the heart, which must pump harder against resistance, leading to the thickening of the heart muscle and potentially decreasing blood flow to the heart. These comorbidities, especially HTN, diabetes, hyperlipidemia, and a family history of coronary artery disease (CAD), are among the most prevalent risk factors for acute myocardial infarction (MI), encompassing both STEMI and NSTEMI [5].

Certain studies have investigated this association, but more precise insights are pivotal. Therefore, we sought to better understand the relationship between the impact of various comorbidities on the outcomes in NSTEMI patients, which would also help physicians develop more effective treatment strategies. Our study aims to find an association between these comorbidities and NSTEMI patients, which could improve patient outcomes and advance medical knowledge.

## Review

Pathophysiology

ACS includes a range of conditions related to a sudden reduced blood supply to the heart, including unstable angina, NSTEMI, and STEMI [6]. Classification of NSTEMI and STEMI is based on their electrocardiographic presentation; STEMI presents with a ≥2mm ST-segment elevation and prominent T-waves on the electrocardiogram, whereas NSTEMI depicts an ST-segment depression and prominent T-wave inversion on the electrocardiogram [7].

The underlying pathology is associated with increased demand due to thrombotic vessel occlusion [6]. More than 90% of cases of coronary syndromes involve an atherosclerotic plaque rupture or erosion [8]. Immunity and inflammation lead to plaque growth [8]. Upon rupture of the plaque, a thrombogenic core is exposed, which then triggers platelet aggregation and thrombus formation, thereby reducing blood flow to the myocardium [9].

The lack of blood flow results in a hypoxic state, which triggers a cascade of cellular events that affect the heart's ability to correctly utilize aerobic metabolism for energy production. This leads to cellular acidosis, which not only disrupts cellular function but also impairs contractility.

**Figure 1:**
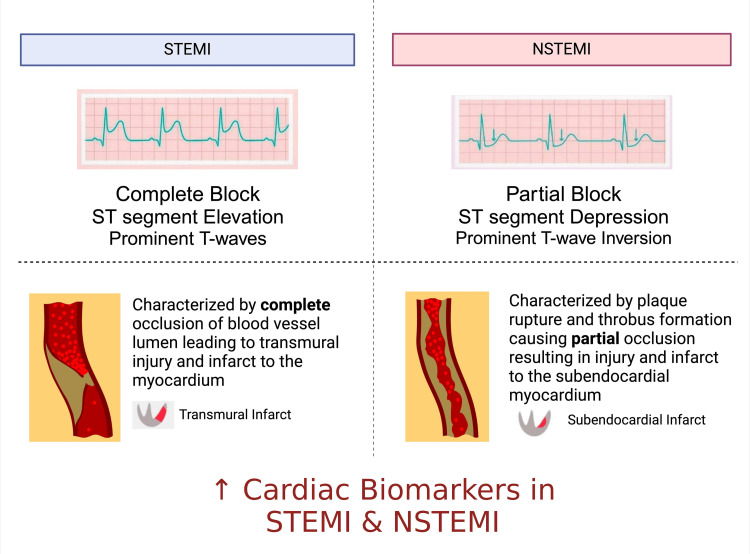
Overview of STEMI vs NSTEMI. Created using biorender.com. NSTEMI: Non-ST Elevation Myocardial Infarction; STEMI: ST-segment elevation myocardial infarction.

Several other factors can worsen NSTEMI, including diabetes due to microvascular dysfunction or other inflammatory comorbidities like obesity, smoking, and HTN, which can accelerate atherosclerosis and plaque vulnerability [10]. Figure 1 below shows an overview of the differences between the two MIs.

Diagnosis

The main diagnostic tests are past medical history, ECG, and cardiac biomarkers.

ECG is the most commonly used initial test for evaluation and should be performed immediately in patients with chest pain or ACS [10]. ST-segment depression is usually seen in NSTEMI, unlike STEMI, which presents with ST-segment elevation.

The most sensitive and specific markers for myocardial injury are cardiac troponins. Troponin is released upon cell death due to the rupture of cell membranes, causing intracellular contents to spill into the extracellular space, eventually reaching the bloodstream [11]. Once troponin is spilled in large enough quantities, it can be detected in circulating blood. Troponin levels usually elevate within 2-3 hours of the onset of chest pain and continue to rise until a peak is reached, generally between 12 and 48 hours [12].

The only drawback is that elevated levels of troponin cannot define the cause of the injury, and hence, in clinical work, it may be difficult to interpret the variation in the level of troponin in conditions such as stroke, pulmonary embolism, sepsis, and other pathologies [13].

Standard Treatment Protocols

NSTEMI requires immediate and standardized treatment to reduce myocardial injury as well as to improve patient outcomes. Treatment and management of NSTEMI are well-defined in international guidelines, with a focus on optimum medical therapy, revascularization procedures, and risk stratification [14,15].

Pharmacological treatment for certain conditions includes several components. Dual antiplatelet therapy typically includes aspirin combined with a P2Y12 receptor blocker such as clopidogrel [14,15]. For patients experiencing ongoing ischemia or those planned for invasive procedures, heparin or low-molecular-weight heparin (LMWH) can be utilized [14]. Beta receptor blockade, using agents such as metoprolol, is also commonly used to reduce heart rate and myocardial oxygen demand [14]. In patients with heart failure or diabetes, ACE inhibitors or angiotensin receptor blockers are prescribed [14]. Additionally, statins are used to prevent future cardiovascular events in patients with high cholesterol [14].

Individuals with high-risk features such as ongoing ischemia, hemodynamic instability, and significant ST-segment changes often benefit from an early invasive strategy with percutaneous coronary intervention (PCI) to open clogged arteries. In contrast, individuals with low-risk features based on clinical presentation and biomarkers are managed with close monitoring and delayed revascularization based on stress testing and symptom recurrence [14].

**Figure 2:**
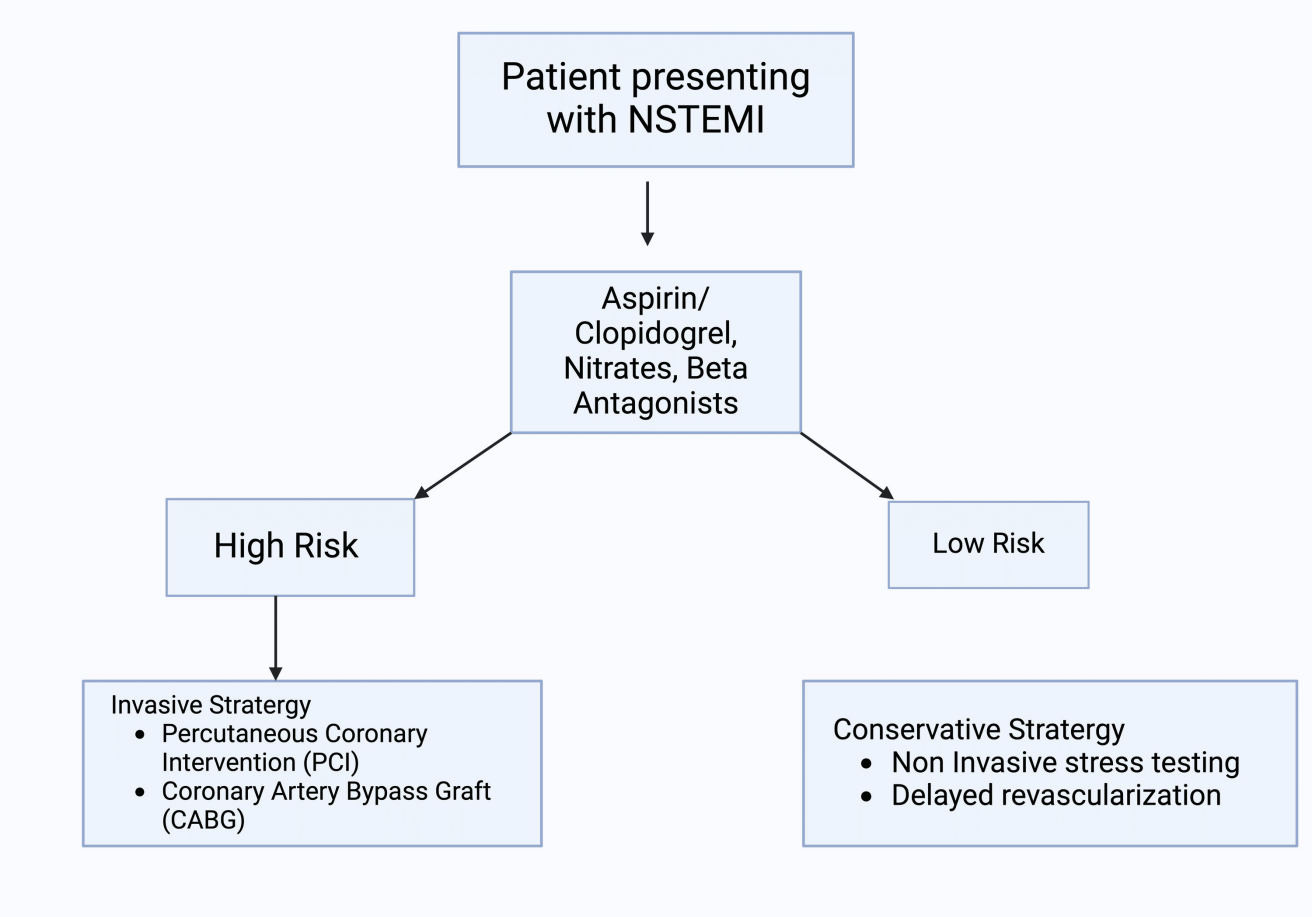
Overview of treatment protocols for NSTEMI. NSTEMI: Non-ST Elevation Myocardial Infarction

Revascularization procedures are done to improve blood flow to the heart muscle and can be either performed via PCI, which involves inserting a stent to open a blocked artery, or coronary artery bypass grafting (CABG), where a healthy blood vessel is grafted to bypass a blocked coronary artery. Lifestyle modifications such as smoking cessation, a healthy diet, exercise, and weight management also prevent accelerated cardiovascular disease (CVD) [14,15]. Figure 2 shows a diagram overview of treatment protocols for NSTEMI.

Impact of HTN

Arterial HTN, correlating with CAD, is one of the world's most significant cardiovascular risk factors [16]. Numerous pathophysiological connections have been discovered between ACS and HTN. Through various pathways, including G protein-coupled adrenergic receptors, endothelial dysfunction, sympathetic vasoconstriction, and vascular wall hypertrophy, it has been shown that sympathetic hyperactivity contributes to the atherosclerotic process in hypertensive individuals [17]. A higher risk of ischemia and arrhythmic episodes eventually develops due to the direct sympathetic action on the renin-aldosterone axis, leading to the development of left ventricular hypertrophy [18]. Catena et al. [18] also suggest the existence of a prethrombotic state in hypertensive patients, including elevated platelet aggregation and activation of clotting proteins like fibrinogen [18]. Raised blood pressure indicates more mechanical strain on blood vessels, exacerbating endothelial dysfunction, accelerating the development of atherosclerosis, and ultimately leading to plaque rupture [19,20].

HTN is the primary modifiable risk factor in CVDs, and its prevalence is increasing [20]. Mills et al. [21] reported that in 2010, HTN affected 31.1% of the world's adult population (1.39 billion people). It is defined as systolic blood pressure of at least 140 mmHg and diastolic blood pressure of at least 90 mmHg [21].

CAD is found to be significantly increased in patients with HTN [22]. A study reported that young individuals with CAD had a 25% prevalence of HTN, whereas young individuals without CAD had a 13% prevalence [22]. Reinstadler et al. [23] reported that the prevalence of HTN increases to 70% in patients with NSTEMI and is between 30% and 40% in those with STEMI [23]. According to AHA statistics from the Coronary Artery Disease national registry, 63.1% of patients with NSTEMI in the population analyzed between 2007 and 2008 also had HTN [24,25]. In another study, systemic arterial HTN was observed in 63% of patients less than 40 years of age with ACS [26].

It was proposed that HTN potentially impacts the prognosis in patients with NSTEMI independent of the treatment modality given. Saluveer et al. [26] studied 175,892 patients for post-PCI outcomes, out of which 78,100 had a history of HTN. Acute MI led to PCI in 78,100 (71%) of the hypertensive patients. The highest risk in STEMI and an intermediate risk in NSTEMI were linked to HTN. Patients with high blood pressure following an acute MI may experience accelerated and worsened post-infarction cardiac remodeling [26]. Experimental results show that myocardial thinning and infarct enlargement are made worse by a brief, moderate post-infarction pressure rise, which can have long-term effects on the ventricles' size, shape, and prognosis [27,28].

Opposite conclusions were observed in several other studies. Cecchi et al. [28] investigated the distinct impact of HTN on prognosis between 437 patients treated with PCI for NSTEMI and 1031 patients treated with STEMI. In either group, HTN was not linked to either long-term or in-hospital mortality [28]. Abrignani et al. [29] examined 4994 MI patients and found that ventricular fibrillation, cardiac rupture, atrioventricular block, cardiogenic shock, and ventricular thrombosis were less common in HTN patients. Regardless of the location of the infarct, normotensive patients had a significantly higher in-hospital death rate than hypertensive patients [29].

Despite being the primary risk factor for NSTEMI, HTN may also have a cardioprotective effect in certain situations, such as during the acute phase of an MI, when individuals with HTN seem to have a better in-hospital prognosis [30]. There is a possibility that individuals with admission systolic blood pressure of more than 110 mm Hg and diastolic blood pressure of less than 70 mm Hg before discharge have a better long-term prognosis. In any case, it should be noted that in order to minimize subsequent long-term adverse events, adequate long-term blood pressure control is required [31].

HTN interaction in NSTEMI protocol

HTN can predispose to the formation of atherosclerosis, incidence of peripheral and CAD, chronic renal failure, heart failure, and cardiovascular death. Emanuele Cecchi et al. [28] studied the prognostic impact of HTN in 1,031 STEMI patients and 437 NSTEMI patients treated with PCI for 40.2 months and found that HTN was higher in NSTEMI compared to STEMI patients (p < 0.001) [28].

HTN also plays a vital role in the management of NSTEMI. It is one of the three risk factors mentioned in the Thrombolysis In Myocardial Infarction (TIMI) score, considered a tool for early risk stratification [32]. The TIMI risk score also helps decide the next steps in the NSTEMI approach. It consists of seven factors including age more than ≥ 65 years, presence of risk factors, presence of ≥ 2 episodes of angina 24 hours before presentation, ST deviation more than or equal to 0.05mV on initial ECG admission, elevated cardiac markers, previous history of coronary artery stenosis ≥ 50%, and usage of aspirin in the past seven days.

Each factor adds a point toward the TIMI risk score, leading to a total possible score of 0 to 7. Subsequently, the approach is based on the score after the initial management of NSTEMI (aspirin - clopidogrel - heparin - beta blockers - nitroglycerin - statin). A TIMI score of 5 and above requires coronary angiography [33]. It must be acknowledged that blood pressure below 90/50 (shock) can be a sign of instability in ACS patients and calls for urgent coronary angiography [33]. Thus, maintaining blood pressure in the 120-140 range as part of the management of NSTEMI cases is associated with a good prognosis [34].

Cecchi E. et al. showed fewer antihypertensives in NSTEMI cases than in STEMI upon admission. However, at discharge, all patients in both groups had taken antihypertensives [32]. In literature, starting with sublingual nitroglycerin, away from being used in controlling the pain in NSTEMI, it has an excellent antihypertensive part [35]. The intravenous type is used if there is no relief or pain, and blood pressure should be titrated until normalized in hypertensive patients unless unwanted side effects appear [36]. Nitrates are contraindicated in patients with recent phosphodiesterase intake. If nitrates are contraindicated, morphine can be used to manage the pain symptoms and decrease blood pressure. However, it should be used cautiously as there is debate about its effect on ticagrelor [36]. Nitrates should be followed with oral beta blockers, with or without angiotensin-converting enzyme inhibitors [35]. However, there is a recommendation against beta blockers as they can be harmful when a patient is in shock. Beta blockers' effect on blood pressure helps reduce oxygen demand by myocardial muscles, and they are very beneficial over the long term post-NSTEMI. From the beta blocker family, the B1 family is the preferred one (cardioselective).

Moreover, ACEs also reduce mortality in NSTEMI. So, unless contraindications exist, it can also be prescribed at discharge [35]. Continuing on medication is essential, as it is found that for long-term adverse effects for further cardiovascular issues, HTN was a dependent prognostic indicator according to data ( OR 0.83, 95% CI 0.50 - 1.39, p = 0.481) [32]. Adherence is a big challenge; it is total team management, starting from teaching patients about the pills, the next time to dispense, and many others. Moreover, this does not mean it will always lead to good patient results [35].

More advances on NSTEMI medications and interaction with high blood pressure management (HTN) in accordance with the AHA: One of the indications for intravenous nitroglycerin during the NSTEMI is HTN. However, if a patient recently received a phosphodiesterase inhibitor, nitrates become contraindicated at the moment [36,37]. Again, beta-blockers should be started as soon as possible if the patient does not have any signs of shock, heart failure, heart block, or symptomatic asthma status [35,36,37]. Even if a patient is contraindicated for beta blockers, it should be reevaluated later so the patient can resume it for patients experiencing recurrent ischemia in NSTEMI who have contraindications to beta blockers. Calcium channel blockers (non-dihydropyridine type) should be given if the patient is not on decompensated left ventricular dysfunction, shock, or prolonged PR interval. For ACE inhibitors, they should be used life-long unless contraindicated in patients with comorbidities like HTN. Angiotensin receptor blockers are recommended for patients who cannot tolerate ACE inhibitors [37].

HTN plays a crucial role in CAD. Because of the existence of PCI, HTN does not affect prognosis at the time being, but still, HTN should be strictly controlled after a MI episode as it will help prevent further cardiovascular events [32].

Impact of diabetes on NSTEMI

Diabetes mellitus (DM) occurs due to chronic hyperglycemia, resulting from insufficient insulin secretion, diminished response, or both. It can be triggered by genetic predisposition, an unhealthy diet, a sedentary lifestyle, and obesity, leading to metabolic dysfunction affecting carbohydrates, fats, and proteins [38].

Approximately 463 million people worldwide were estimated to have DM in 2019, representing 9.3% of the global population. This number is projected to increase to 578 million (10.2% of the global population) by 2030 and 700 million (10.9% of the global population) by 2045, making it a critical comorbidity to address [39]. Type 1 diabetes results from the autoimmune destruction of pancreatic islet cells that produce insulin. Type 2, the more prevalent form constituting 90% of all diabetes cases, is characterized by insulin resistance and β-cell dysfunction [40].

Glycated hemoglobin (HbA1c) is the gold standard for assessing glycemic status, representing the average blood glucose level over the past 4 to 8 weeks. The American Diabetes Association (ADA) stratifies glucose metabolism based on HbA1c as follows: normoglycemia (NG, HbA1c < 5.7%), pre-diabetes (pre-DM, 5.7% ≤ HbA1c < 6.5%), and diabetes (DM, HbA1c ≥ 6.5% or diagnosed DM) [41]. Chronic complications associated with DM include macrovascular conditions such as stroke, CAD, peripheral arterial disease, and microvascular conditions, including diabetic kidney disease, retinopathy, and peripheral neuropathy [42]. The risk posed by type 2 DM is considered equivalent to that of nondiabetic patients with known atherothrombosis [43]. MI is the predominant cause of death in these patients, who are more prone to present with NSTEMI and have significantly higher all-cause mortality compared to those with STEMI. The likelihood of developing MI within ten years of developing type 2 diabetes is more than 20%, with a greater than 40% chance of recurrence in the future [44,45].

DM significantly contributes to the formation of atherosclerotic lesions and thrombi in NSTEMI patients due to atherogenic risk factors they possess, such as HTN, obesity, dyslipidemia, and insulin resistance. Long-term hyperglycemia, insulin resistance, and increased fatty acids induce adverse metabolic alterations within the endothelium that contribute to endothelial dysfunction, increased coagulability, elevated platelet aggregation, and fibrinolytic impairment leading to extensive CAD (CAD), and a higher rate of multi-vessel involvement. Furthermore, lipid and atheromatous plaques with intense macrophage infiltration, high pro-inflammatory cytokines (MCP-1, IL-1), and matrix metalloproteinases lead to decreased synthesis and increased breakdown of collagen in the fibrous cap, making it vulnerable to rupture and thrombosis [46].

DM escalates the incidence of silent myocardial ischemia, meaning the patient presents without chest pain or associated symptoms like dyspnea, palpitations, nausea, and diaphoresis. Cardiac autonomic dysfunction involving the pain receptors, afferent neurons, or higher brain areas is responsible for this. Older patients with DM and those with a history of MI or revascularization are particularly susceptible. These silent infarctions, or infarctions with atypical symptoms, prolong the time to hospital admission and diagnosis [47]. Additionally, NSTEMI patients have no ST-segment elevation, which further delays diagnosis. All these factors collectively contribute to the delay in reperfusion to the culprit artery within the optimal therapeutic period, resulting in extensive infarction and a higher mortality rate [44]. Studies show that diabetic MI patients had significantly higher levels of total cholesterol, creatinine, BUN, and potassium on admission, as well as a higher chance of fatal in-hospital outcomes such as atrial fibrillation, cardiogenic shock, heart failure, and stroke [43].

Although revascularization following an acute episode significantly reduces the risk of mortality, a more conservative approach of coronary artery bypass grafting (CABG) is often recommended in NSTEMI patients with diabetes. Underlying reasons include incompatible coronary anatomy due to extensive CAD, the risk of restenosis, and the higher prevalence of renal dysfunction in these patients, restricting the use of contrast media. However, drug-eluting stents and adjunctive therapy with glycoprotein IIb/IIIa inhibitors during the procedure have been shown to improve the outcome after percutaneous coronary intervention (PCI) in these patients [48].

Diabetic MI patients are more susceptible to adverse outcomes than their nondiabetic counterparts. The severity of CAD plays a pivotal role in determining long-term survival in these patients. Treatment strategies should focus on preserving and optimizing myocardial function, stabilizing vulnerable plaques, and preventing recurrent events. For effective long-term management, diabetic patients with CVD should undergo comprehensive risk factor management, including glycemic control, control of blood pressure, and lipid levels. They should receive effective anti-platelet therapy regimens and lifestyle interventions [46,47].

Impact of CKD

Patients with chronic kidney disease (CKD) have worse outcomes related to CAD [49]. In patients with MI, CKD is more prevalent among those with NSTEMI and is associated with increased in-hospital bleeding and mortality. It is also shown that there is an inverse association between the incidence of adverse outcomes and kidney function [50]. The unfavorable outcomes in CKD patients who have CAD could be explained by their receiving less invasive treatment strategies, such as percutaneous coronary intervention (PCI). This was demonstrated in a study enrolling patients from the National Cardiovascular Data Registry (NCDR) ACTION-GWTG registry from January 1, 2007, to December 31, 2009, where only 30% of NSTEMI patients with CKD underwent PCI [51].

The ACTION-GWTG Registry studied how cardiac biomarkers, primarily troponin and creatinine kinase MB, affected the treatment regimen in patients with non-ST elevation myocardial infarction [52]. Since there were differences in pharmacological treatment between troponin-positive and creatinine kinase MB-negative (Tn(+) and CKMB(-)) NSTEMI patients, it mainly focused on these patients [52]. Patients with NSTEMI who had known CAD, revascularization, or previous MI and lacked biomarker data were excluded. Of the 16,064 patients examined, 28% had CKMB (-), and 72% had CKMB (+). Treatments and hospital-based analyses were performed, and the results indicated that the CKMB (-) patients had higher comorbidities, such as diabetes (31% vs. 27%), HTN (71% vs. 66%), and were currently on dialysis (2.5% vs. 2.0%), and they were also older (68 vs. 65) [52].

There was also a difference in the first-day treatments that these patients received in the hospital, such as antithrombins, clopidogrel, glycoprotein 2b/3a, and even invasive procedures like revascularization (51% vs. 55%) and coronary angiography (40% vs. 54%) [52]. After correcting for baseline factors, the hospital mortality rate for CKMB (-) patients was relatively low despite all the variations. Accordingly, this study concludes that despite significant risks such as advanced age and increased comorbidities such as heart failure, renal failure, and others, the Tn (+) and CKMB (-) NSTEMI groups are primarily disregarded and receive inadequate treatment [53]. Therefore, it is necessary to focus more on enhancing the quality of life for CKMB (-) patients [52].

The three large randomized trials, namely FRISC-2, RITA-3, and ICTUS, endorsed that invasive treatment improves outcomes in patients with NSTEMI. Still, there is less evidence regarding its implementation in the elderly comorbid NSTEMI group [53]. To evaluate its effectiveness, a clinical trial was conducted by MOSCA, which included 106 elderly (age >70) comorbid (like renal failure, peripheral artery disease, COPD, anemia, dementia) NSTEMI patients divided almost equally into two groups - invasive and conservative [53].

The most prevalent comorbidity in both of these groups was renal failure, and there were many other commonalities as well, such as the kind of medical care provided both during admission and after discharge [53]. Conversely, the rates of cardiac catheterization and revascularization were 100% and 20%, 58% and 9%, respectively, when it came to invasive care. There was very little statistical significance in the death rate of 42% vs. 48% following a series of cumulative events (bleeding, reinfarction, and post-discharge revascularization) during the trial [53]. The invasive strategy had better outcomes in the first three months but later vanished in the long term. However, this should not forbid elderly comorbid NSTEMI patients from having invasive management [53].

Despite the recommendations of the ACC/AHA and the European Society of Cardiology guidelines on an urgent invasive strategy in high-risk patients presenting NSTEMI, most patients are deferred because of the risks of acute kidney injury accelerating their progression to dialysis. On a further note, the bleeding risk requiring transfusion had an incremental increase in NSTEMI patients with CKD when compared with no CKD by 20% in stage 3, 48% in stage 4, and 59% in stage 5 [54].

**Figure 3:**
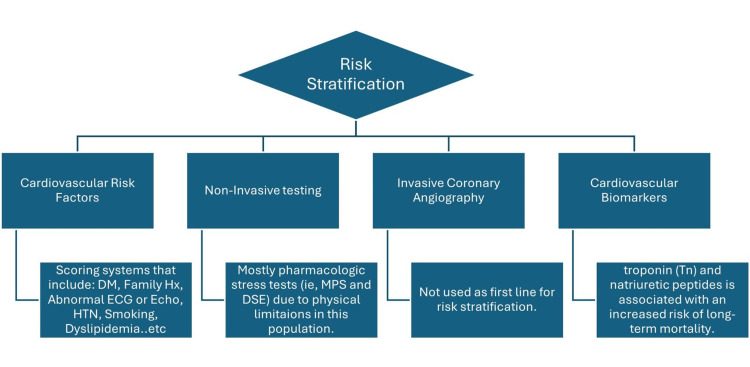
Overview of risk stratification to predict outcomes in patients with renal impairment. DM: Diabetes Mellitus; HTN: Hypertension; MPS: Myocardial Perfusion Scintigraphy; DSE: Dobutamine Stress Echocardiography.

The risk-to-benefit ratio needs to be assessed for every NSTEMI patient with CKD to undergo invasive management as its utilization decreases mortality but increases the risks of having in-hospital acute kidney injury requiring dialysis and blood transfusion. Randomized trials need to be improved in this regard [55]. Clinicians are forced to extrapolate evidence from observational studies, expert opinion, and deductions from the general population to risk stratify and predict the best outcome for these patients. Although there are variations in published recommendations, clinical scoring systems represent patients’ comorbidities and functional status, as shown in the figure below (Figure 3) [56,57].

The higher mortality rate could also be explained by NSTEMI patients typically having a greater number of comorbidities, which is attributed to increased inflammation and oxidative stress. Roumeliotis et al. [55] showed that low ejection fraction (EF) after NSTEMI-ACS was associated with inflammatory markers such as hs-CRP, IL-10, IL-6, and MPO. Regarding kidney function, BNP, IL-10, and TGF-β increased significantly across albuminuria categories with a statistically significant difference [55].

Impact of COPD

Chronic obstructive pulmonary disease (COPD) is characterized by airflow limitation and is typically caused by chronic bronchitis or emphysema. Chronic bronchitis involves the narrowing of the airways due to inflammation and excessive mucus production, while emphysema results in a loss of elasticity and impaired gas exchange.

Recent research indicates that COPD significantly affects the cardiovascular system, especially in the Western world, where it is now one of the leading causes of myocardial infarction in that population [58]. Smoking, a major contributor to the development of COPD, also promotes the development of atherosclerosis, affecting blood vessels and leading to CVD [59].

One objective of our study was to demonstrate how comorbidities affecting the lungs impact the heart and contribute to the development of NSTEMI. Our analysis revealed that most previous studies discuss the close relationship between the heart and lungs, as they share common risk factors such as smoking and aging. Furthermore, COPD patients tend to have higher rates of other heart disease risk factors like diabetes and HTN when compared to healthy individuals. Studies have found that 10% to 38% of COPD patients have CVD, and people with COPD, especially those in younger age groups, are more prone to developing acute MI as they age [60].

Mechanism of action

**Figure 4:**
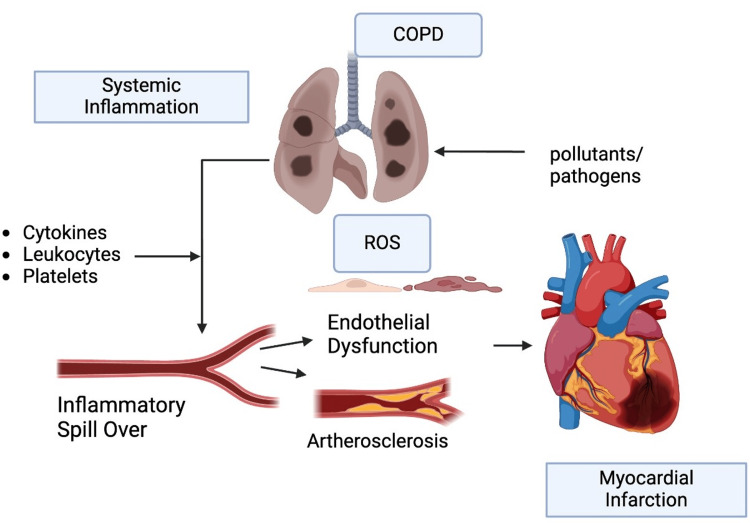
Inflammation progressing to plaque formation and eventually leading to MI. Created using biorender.com MI: Myocardial infarction; COPD: Chronic obstructive pulmonary disease; ROS: Reactive Oxygen Species.

COPD triggers a series of inflammatory processes in the lungs and induces a systemic response, leading to plaque formation in the arteries. This inflammation can also elevate levels of specific proteins involved in blood clotting, such as interleukin-6, C-reactive protein, and fibrinogen. These proteins promote the formation of blood clots and, in turn, increase the risk of thrombotic events like stroke or infarctions, as demonstrated in the figure below (Figure 4) [58].

Presentation in patients with COPD

Studies conducted in the past show that patients suffering from COPD are more likely to present with breathlessness, atypical chest pain, and palpitations rather than typical chest pain [60,61,62]. Research indicates that COPD patients are more likely to have NSTEMI than STEMI compared to those without COPD. Additionally, they were found to have low levels of diagnostic biomarkers such as troponin and creatinine kinase [60].

The prognosis after an acute MI for those with COPD is much worse than for those without COPD [59]. A study conducted on 9,158 patients admitted to Tehran Heart Centre (THC) with NSTEMI between September 2003 and April 2017 also indicated that research done in the past demonstrated that patients with chronic lung disease presenting with NSTEMI had a higher risk of in-hospital mortality, which was probably due to an increased risk of bleeding and different treatment approaches. In the study, 3,133 of them had type 2 DM, and 57% of the study population were men. It was found that 78.8% was due to cigarette smoking, making it the most common risk factor. The study also showed that patients with DM were either managed with an oral hyperglycemic agent, which was common among the population (59.9%), and less commonly with insulin (20.9%). Out of 117 patients who died during admission, 47% were women. The prevalence among COPD participants who took part in the study was very low, at 1.4%. However, the prevalence of COPD among patients who died was much higher at 3.4%. Zhang et al. [63], conducted a study that evaluated patients who underwent percutaneous coronary intervention (PCI), and this study concluded that COPD was strongly linked to CVD leading to death [63].

The outcomes after a heart attack, particularly in those patients with COPD, tend to show an increased incidence of risk for other complications, such as heart failure, both in the patients and after discharge from the hospital. Interestingly, studies show that those with COPD did not have a higher risk for the development of atrial fibrillation, cardiogenic shock, or stroke but were more likely to develop acute heart failure [60]. A nationwide study conducted recently in Taiwan included patients with acute MI (AMI) and concomitant COPD, aged between 18-120 years, hospitalized between January 2000 and December 2012. The study population was then divided into STEMI and NSTEMI groups. Out of 186,326 patients hospitalized with AMI, 214 were excluded based on their exclusion criteria. The remaining 186,112 patients were included in the study, and 112,408 had NSTEMI. The 12-year follow-up concluded a 78.99% mortality among patients with NSTEMI and COPD and 70.52% mortality among NSTEMI patients without COPD. Following the study, it was discussed that post-AMI medications, if administered, can reduce the mortality rate in all patients with AMI regardless of COPD status. Another sizable retrospective study done in the UK showed that in-hospital and 180-day mortality was common in patients with AMI and COPD. The study showed a 26% higher incidence of mortality in patients with AMI and concomitant COPD than in those with AMI without COPD [64,65].

From our analysis of COPD as a comorbid condition for patients with NSTEMI, we can conclude that the heart and lungs are interlinked and tend to interact in terms of disease, especially since they share common risk factors such as smoking and aging [58,59].

Impact of obesity

In recent decades, there has been a dramatic increase in people with obesity worldwide. Obesity is an independent risk factor for all types of heart disease, including heart failure, high blood pressure, ischemic heart disease, and atrial fibrillation [66]. Unhealthy lifestyle factors such as smoking, overweight, sedentary lifestyle, type 2 DM, and HTN are known to contribute to the global epidemiological pattern [66,67]. Obesity is characterized by an increased BMI [68]. BMI is a numerical value calculated based on weight and height and is primarily used as a screening tool to assess the risk of weight-related health conditions [69].

The BMI defines 18.5-24.9 as healthy weight, a BMI of ≥25 to <30 kg/m2 as overweight, and a BMI of ≥30 kg/m2 as obesity [70]. Individuals with a typical normal BMI are susceptible to possessing moderate levels of adiposity and maintaining metabolic health [70]. Conversely, those with a high BMI exhibit high levels of adiposity and are prone to develop obesity-related ailments [70].

There are metabolically healthy and unhealthy individuals; however, many individuals classified as obese present minimal or no elevated metabolic risk factors as defined by the metabolic syndrome, suggesting a subgroup termed "metabolically healthy obese" who may not be susceptible to cardiovascular risk [66,67]. Even though evidence is lacking regarding the criteria defining this subgroup, estimates indicate that 3 to 57% of obese individuals qualified for this category [65,71]. Nonetheless, studies have demonstrated that people who fall into this subgroup will progress to metabolically unhealthy obesity due to the prevalence of obesity-related comorbidities such as sleep apnea, gestational HTN, PCOS, osteoarthritis, airway hyperresponsiveness, and psychological distress [65,71]. In contrast to unhealthy obese individuals, metabolically healthy obese individuals present with reduced visceral mass and decreased fat accumulation in the liver and muscle, decreased macrophages and other immune cells in adipose tissue, and reduced inflammation [65,68,71]. Metabolically healthy obese subjects demonstrate a favorable inflammatory status.

The prejudicial impacts of obesity may also be influenced by environmental, biological, and socioeconomic factors [65]. Obesity has been one of the most significant nutritional disorders and a major risk factor for NSTEMI [72]. Obesity contributes to cardiovascular risk factors such as dyslipidemia, insulin resistance, HTN, and DM, all of which play a role in the pathogenesis of NSTEMI [65,73]. It has been found that one of the molecular mechanisms involving early life obesity includes epigenetic modifications of genes, which consist of methylation, histone modification, chromatin remodeling, and alterations in non-coding RNA [65,68,72]. These changes increase the risk of developing adult obesity and can be inherited by future generations [65,73]. Elevated abdominal adiposity may contribute significantly to metabolic and vascular diseases [68,72]. Adipocytes synthesize adipokines whose secretion rates are influenced by the amount of adipose tissue present [73]. Adipocytes secrete leptin, resistin, and cytokines such as tumor necrosis factor (TNF) [65,72,73], therefore, adipose tissue secretes a pro-inflammatory state, predisposing individuals to CVD [71,73]. Leptin plays a role in regulating appetite. Studies have shown that increased leptin levels in obese individuals signal leptin resistance [71]. Resistin levels also increase in obesity, both of which have a role in the mechanism of inflammation, which can lead to CVD [65,72]. Therefore, resistin and leptin, known for their role in inflammation, increase C-reactive protein (CRP) levels as a significant inflammatory marker [71,73]. The increased marker stimulates endothelial cells to produce more CRP, which promotes vascular thrombosis, contributing to the pathophysiological process of CVD [65,71,72,73].

Metabolic syndrome, characterized by hyperglycemia, elevated triglycerides, HTN, obesity, and low high-density lipoprotein levels, is a risk factor for CVDs [74,75]. Previous studies have explored the link between MI and metabolic syndrome, indicating that metabolic syndrome is a risk factor for MI. A meta-analysis was conducted to evaluate how metabolic syndrome relates to MI in individuals with excess body weight [74]. The study design was structured by individuals classified with excess body weight; the intervention and exposure involved diagnosing metabolic syndrome; the comparison group consisted of individuals with an average BMI, and the outcome was MI, with a total of 61,104 participants from eight different regions such as Japan, Australia (was included twice by different authors, Leon Simons and J. Opio), United States, Denmark, Korea, Europe, and China [75]. They included nine studies in the analysis, including those by Ogorodnikova et al. Studies of obese participants with a BMI of 33.7 kg/m2, while Lee et al. focused on overweight [74]. The overall meta-analysis results showed an association between metabolic syndrome and MI among obese individuals [75]. It was perceived as a notable positive link between metabolic syndrome and MI among obese patients. The overall findings of nine studies were analyzed. Only one reported a significant inverse relationship between metabolic syndrome and MI [75].

Impact of dyslipidemia

According to Hedayatnia et al. [74], dyslipidemia refers to elevated levels of low-density lipoprotein cholesterol (LDL-C) ≥ 130 mg/dL, triglycerides (TG) ≥ 150 mg/dL, or a reduced concentration of serum high-density lipoprotein cholesterol (HDL-C) < 40 mg/dL. Berberich and Hegele [75] state that the criteria for dyslipidemia are based on specific lipid level thresholds, which help identify individuals at risk for CVDs due to lipid abnormalities.

Muneeb et al. [76] note that dyslipidemia is a significant comorbidity observed in patients with NSTEMI. In a study of 101 individuals with ACS, 27 of whom were diagnosed with NSTEMI, it was found that 74.1% of NSTEMI patients had dyslipidemia. The lipid abnormalities identified included elevated triglycerides (53.5%), high LDL cholesterol (50.5%), low HDL cholesterol (72.3%), high total cholesterol (15.8%), and abnormal VLDL levels (50.5%). This high prevalence emphasizes the crucial importance of effective dyslipidemia management in reducing cardiovascular events in NSTEMI patients. It underscores the significance of early detection and intervention to address lipid abnormalities and associated cardiovascular risks [76].

In a recent investigation conducted by He et al. [77], 315 pairs of age- and sex-matched CAD and non-CAD subjects were examined to investigate the impact of hypersensitive C-reactive protein (hs-CRP) on the association between dyslipidemia and CAD. The findings demonstrated that both dyslipidemia and increased hs-CRP levels significantly raised the likelihood of CAD. Furthermore, a positive correlation was observed between dyslipidemia and elevated hs-CRP levels. Through mediation analysis, it was determined that 8.27% of the link between dyslipidemia and CAD was influenced by hs-CRP levels, suggesting that inflammation, as indicated by hs-CRP, plays a partial role in the development of CAD in the presence of dyslipidemia. This underscores the importance of considering lipid levels and inflammatory markers in managing and understanding CAD [77].

The 2020 guidelines by the American Association of Clinical Endocrinologists (AACE) and the American College of Endocrinology (ACE), as outlined by Handelsman et al. [78], focus on comprehensive dyslipidemia management and CVD prevention. They recommend starting with lifestyle changes such as healthy eating, regular exercise, and weight management. For those at extreme risk of atherosclerotic cardiovascular disease (ASCVD), the guidelines suggest high-intensity statin therapy to lower LDL-C levels to less than 55 mg/dL. Very high-risk individuals should aim for levels under 70 mg/dL, while moderate-risk patients should strive for levels below 100 mg/dL. Combining therapies, such as adding ezetimibe or PCSK9 inhibitors, is suggested for those who do not reach LDL-C targets with statins alone. The guidelines also provide specific recommendations for managing triglycerides and other lipid abnormalities, including high lipoprotein(a) levels [78-80].

Smoking

Smoking can eventually lead to ACS, including NSTEMI, through a pathological process known as atherosclerosis of blood vessels [81]. It is one of the modifiable risk factors that predominantly affects men younger than women, leading to a high mortality rate in them [82].

Atherosclerosis leads to the formation of either stable or unstable plaque. Unstable plaque formation is mostly seen in smokers because nicotine, a component of cigarettes, induces the production of metalloproteinases 2 and 9 (MMP2 & MMP9) through a sequence of events that are responsible for unstable plaque formation [83]. It has been proven that smoking cessation can reverse the pathological sequence of atherosclerosis in the long term and also prevent further attacks [81].

Numerous studies have demonstrated that smokers had better clinical outcomes following an acute MI despite the deleterious consequences of smoking on cardiovascular health. This finding suggests a phenomenon known as the 'smoker's paradox.' According to these studies, smoking preconditions the heart cells for ischemia, which stops subsequent assaults. However, research indicates that the smoking paradox may not be entirely justified because age was found to be a significant factor in both smokers' and non-smokers' clinical outcomes, with smokers being somewhat younger and having fewer comorbidities [84].

Smoking also plays a significant role in patients who are discharged after a MI following percutaneous coronary intervention (PCI). Although the immediate mortality rate is similar in both STEMI and NSTEMI smokers, there is a significant difference in mortality rate in the later years in which NSTEMI smokers take the lead [85].

Being the most crucial risk factor in causing acute MI, smoking cessation has been known to decrease the mortality rate in post-discharge patients. Smoking cessation is one of the most challenging tasks to achieve, but with timely interventions and proper hospital counseling, it is known to be effective [86].

## Conclusions

In conclusion, the presence of comorbidities in patients with NSTEMI significantly influences their clinical outcomes and management strategies. This narrative review has discussed how comorbid conditions such as diabetes, HTN, CKD, and heart failure complicate clinical presentations and heighten the risk of adverse events, including mortality and recurrent cardiovascular incidents. The impact of comorbidities on patients with NSTEMI necessitates a tailored therapeutic approach that extends beyond standard protocols, emphasizing the importance of comprehensive risk assessment and individualized treatment plans. Future research should focus on developing and validating predictive models and therapeutic strategies that consider the multifaceted impact of comorbidities to improve the prognosis of patients with NSTEMI.
